# *Tiliroside* suppresses triple-negative breast cancer as a multifunctional CAXII inhibitor

**DOI:** 10.1186/s12935-022-02786-6

**Published:** 2022-11-24

**Authors:** Rui Han, Hongxing Yang, Changquan Ling, Lingeng Lu

**Affiliations:** 1 Department of Chinese Medicine Oncology, The First Affiliated Hospital of Naval Medical University, Shanghai, 200433 People’s Republic of China; 2 Department of Chinese Medicine, Naval Medical University, Shanghai, 200433 People’s Republic of China; 3 Department of Oncology, The First Affiliated Hospital of Guangzhou University of Chinese Medicine, Guangzhou, 510405 Guangdong People’s Republic of China; 4 Department of Chronic Disease Epidemiology, Yale School of Public Health, 60 College Street, New Haven, CT 06510 USA; 5 School of Medicine, Center for Biomedical Data Science, 200 George Street, New Haven, CT 06511 USA; 6 Yale Cancer Center, Yale University, 60 College Street, New Haven, CT 06520-8034 USA; 7 Department of Chronic Disease Epidemiology, Yale School of Public Health, Yale University, 60 College Street, 06520-8034 New Haven, CT USA

**Keywords:** TNBC, CAXII inhibitor, *Tiliroside*, Cancer stemness, Apoptosis

## Abstract

Triple negative breast cancer (TNBC) is an aggressive subtype of breast cancer characterized by poor prognosis, early recurrence, and the lack of durable chemotherapy responses and specific targeted treatments. In this preclinical study, we examines *Tiliroside* (*TS*, C_30_H_26_O_13_), as one of the major compounds of Tribulus terrestris L. which has been used as an alternative therapy in clinic practice of breast cancer treatment, for its therapeutic use in TNBC. The association between *CAXII* expression level and survival probability of TNBC patients, and the difference of *CAXII* expression level between TNBC and normal samples were evaluated by using publicly accessible databases. To determine the anticancer efficacy of *TS* on TNBC cells, cell proliferation, wound healing, cell invasion, and 3D spheroid formation assays were performed and excellent anticancer activities of *TS* were displayed. Mouse models further demonstrated that *TS* significantly reduced the tumor burden and improved survival rate. The properties of *TS* as a novel CAXII inhibitor have also been evaluated by CAXII activity assay, pHi, pHe and lactate level assay. Further RT-PCR and Caspase-3 activity analyses also revealed the positive regulating effects of *TS* on E2F1,3/Caspase-3 axis in TNBC cells cultured in 2D or 3D systems. The findings indicate that *TS* suppresses TNBC progression as a potential novel CAXII inhibitor in preclinical experiments, which warrants further investigation on its therapeutic implications.

## Introduction

Breast cancer ranks the first in the incidence and mortality of malignant tumors in Chinese women, and the incidence is still rising [1, 2]. Breast cancer has undoubtedly become a major threat to women’s health globally. Triple negative breast cancer (TNBC) is a special molecular subtype pattern which is characteristic of negative for estrogen receptor (ER), progesterone receptor (PR) and human epidermal growth factor receptor 2 (HER-2). According to the American Cancer Society, nearly 260,000 women are diagnosed with breast cancer each year, of which 15–20% are TNBC. A larger proportion of younger women is reported with TNBC than older patients [3, 4]. The incidence of breast cancer in young women has increased worldwide in recent years [5]. In the United States, about 11% of breast cancer patients are between the age of 35 and 45 years, and in Asia are 9.5–12%. TNBC is the most aggressive breast cancer subtype in young patients. Compared with other breast cancer subtypes, patients with TNBC had a higher mortality, worse overall survival and higher recurrence rate. Unlike other molecular subtypes of breast cancer, patients with TNBC do not benefit from hormonotherapy and HER-2 targeted therapy. The limited choices of medical treatment for them are usually chemotherapy and immunotherapy but with limited therapeutic effect. Therefore, there is still a tremendous unmet need for the development of novel therapeutics for TNBC [6].

CAXII inhibitor, as a potential cancer treating agent, has been found to impede the creation and transportation of bicarbonate ions into the cells through anion exchangers and Na+/HCO3 − co-transporters, consequently regulating intracellular pH and extracellular acidosis that affect cancer development in the tumor micro-environment [7, 8]. CAXII inhibitor has been thought to improve the apoptosis of cancer cells, but related mechanisms still need to be further explored [9]. In addition, selectively silencing of *CAXII* gene significantly inhibites the migration and invasion of TNBC cells, which ensures the potential of CAXII inhibitor in TNBC treatment [10].

Tribulus terrestris L. (TT), a plant in many regions of Asia and Africa, has been used in traditional Chinese medicine as an herb to treat breast diseases for thousands of years by the formation of oral administration decoction [11]. Nowadays, evidence has proven that the extracts of TT has many medical effects such as antihypertension, diuretic effect and cancer suppression [12]. TT extracts have been found to regulate the apoptosis and metastasis of cancer cells by regulating NF-κB signaling [11]. *Tiliroside* (*TS*, C_30_H_26_O_13_), as a major active ingredient of TT, has been further found to possess anti-inflammatory, anticholinesterase and antioxidant activities [13]. It has been reported to suppress cancer development by regulating NF-κB signaling pathway or MAPK/JNK/p38 axis in leukemia cells [14]. Recently, we further uncovered its anticancer effects on Hepatocellular carcinoma (HCC) cells by targeting CAXII as a novel CAXII inhibitor [9]. One scientific question we asked was whether or not *TS* had the similar anticancer effect against breast cancer.

Thus, the purposes of this study were to investigate the association between *CAXII* expression level and survival probability of TNBC patients, to examine the effects of *TS* on TNBC cells and to explore the related mechanisms in vitro and *in vivo.*

## Materials and methods

### Gene profile overview and survival analysis

Publicly accessed datasets were used to evaluate the prognostic values of CAXII in TNBC and differentiated expression of CAXII. Gene profile overview were conducted in GENTS platform (http://gent2.appex.kr/gent2/) and PEGIA platform (http://gepia.cancer-pku.cn). The Kaplan–Meier survival curves of CAXII in TNBC were generated based on the datasets of triple negative breast cancer RNA-seq using Kaplan–Meier plotter (http://kmplot.com/analysis), in which the optimal cutoff value for CAXII expression was used to classify the patients into two groups, high vs. low CAXII expression.

### Cell culture and transduction of luciferase lentivirus

Human triple-negative breast cancer cell MDA-MB-231, BT-549, and human breast normal epithelial cell MCF-10 A were purchased from American Type Culture Collection (ATCC, USA) and were characterized using STR (Short Tandem Repeat) analysis for identity verification of human cell lines in January, 2022. The cells were cultured in Eagle’s Minimum Essential Medium (EMEM, ATCC, USA), RPMI1640 (ATCC, USA) or Leibovitz’s L-15 medium (LLM, ATCC, USA), respectively, with 10% fetal bovine serum (ATCC, USA), or Mammary Epithelial Cell Growth (MEGM, USA) completed with MEGM bullet kit (Lonza, USA). Cells were cultured in an incubator under the condition of humidified incubator with 5% CO_2_ at 37 °C.

Both MDA-MB-231 and BT-549 cells were transfected with CMV-Firefly luciferase-IRES-Puro lentivirus (Cellomics,USA), and characterized by STR analysis as described previously [15].

### CAXII gene knockout

For establishing CAXII knockout clones, as described in our previous study [9], MDA-MB-231 and BT-549 were both transfected with 1 µg of CAXII CRISPR guide RNA vector plus vector coding for puromycin resistance by applying Metafectene Pro (Biontex, Munich, Germany), respectively. For non-KO clones (as a mock group), CAXII CRISPR guide RNA vector has not been applied. Cells were then selected by puromycin for 16 h. CAXII expression was confirmed after 24 h by RT-qPCR.

### RNA extraction and quantitative RT-PCR

Total RNA was extracted from MDA-MB-231, BT-549 and MCF-10 A cells by using the RNeasy mini kit (Qiagen, Germany) [9]. The concentration and purity of total RNA were determiend by using an Epoch microplate spectrophotometer (Biotek, USA). Total RNA was reverse-transcribed to cDNA templates by using an AffinityScript multi temperature cDNA synthesis kit (Agilent technologies, CA, USA). The expression of detected genes was determined using the SYBR Green PCR Kit (Qiagen, Germany) on a 7500 Fast Real-time PCR System (Life Technologies, USA). All the primer sequences used in this study and qPCR reaction conditions were described in our previous study[9]. Each sample was analyzed in triplicate. After each PCR amplification, dissociation curve was assessed. The relative expression levels of genes were expressed as a fold change relative to *GAPDH* using the 2^−ΔΔCt^ method.

### CAXII activity assessment

CAXII activity inhibition assay was performed following the instructions as described in our previous study [9]. For details, the *TS* was purchased from SIGMA-ALDRICH (USA), the enzyme activity was assessed by monitoring 4-nitrophenol (4-NP/pNP) (SIGMA-ALDRICH, USA). Absorbance of the spontaneous hydrolysis of the substrate alone and substrate treated with *TS* was subtracted from esterase activity in absence and presence of *TS*. In addition, we added potent CAXII inhibitor (U-104) (EMD Millipore corporation, USA) as a positive control. Plotting absorbance (Y-axis) and time (min) (X-axis) was used to evaluate the slope of the initial rate of enzyme activity. The percentage of enzyme activity was then calculated. By applying the non-linear least squares method, the IC50 values were finally calculated.

### Caspase-3 activity

For conducting the CaspACE™ Assay, 2 × 10^6^ MDA-MB-231(or BT-549) cells cultured in 2 mL medium were treated with either 40 µM *TS* or same amount of DMSO as the induced apoptosis group (72 h after intervention), and 3 µL Z-VAD-FMK inhibitor was added in 72 h intervention cells as the inhibited apoptosis groups. The mock groups were a normal control (NC). The plate was incubated at 37 °C in a humidified incubator with 5% CO_2_ for 16 h. After the centrifugation of cell lysates, the cell supernatant fractions were harvested for CASP3 activity measurement. In addition, the protein concentration of each sample was determined by the BCA protein assay (Thermo Fisher Scientific, USA), and the pNA Calibration Curves were made by a colorimetric assay system. CASP3 Specific Activity (SA) was calculated as the formulas as described elsewhere [9, 15].

### Detection of intracellular pH (pHi), extracellular pH (pHe) and lactate level

Intracellular pH was evaluated using the fluorometric intracellular pH assay kit (Sigma, Germany, MAK-150) as previously described [16]. Fluorescent BCFL-AM indicator was used to measure pHi fluctuations in the fluorometric intracellular pH kit for the cells treated by various conditions. The cells were seeded in black-wall plates with 6 × 10^4^ cells in each well, and were incubated for 24 h. The medium was replaced with BCFL-AM reagent prepared in 100 mL of HBS solution (Hank’s buffer with 20 mM HEPES, 5 mM probenecid), then the cells were incubated at 37 °C in an atmosphere of 5% CO2 for 30 min (with avoiding light). *TS* compound at the doses of 0, 20, 40 and 80 µM was added to the HBS solution. After 5 min incubation, the measurement was performed at wavelengths of 490 nm (excitation) and 535 nm (emission) in spectrofluorometry (Spectramax, M5). Extracellular pH (pHe) and lactate levels were evaluated directly using a commercial kit on the blood gas device (ABL90 FLEX PLUS, Radiometer, Copenhagen, Denmark).

### MTS cell proliferation assay

Cell proliferation MTS assay was performed by adding 20 µL of MTS solution (Promega, USA) into each well in a dark hood at different incubation time points (24 h, 48 h, 72 h, 96 h) following the manufacturer’s instruction. Microplate Spectrophotometer (Biotek, USA) was used to detect the cells’ absorbance at the wavelength of 450 nm. Triplicate were conducted for each condition at each time point. The proliferation inhibition rate was calculated based on the formula: inhibition rate = (1-Absorbance of treated sample /Absorbance of control sample)×100%.

### Wound healing assay

MDA-MB-231 and BT-549 cells were cultured in 6-well tissue culture plates with approximately 1 × 10^6^ cells in each well, respectively. Single wound was scratched in each plate when cell monolayer grew to nealry 90% confluence. 40 µM *TS* in DMSO or the same final concentration of DMSO were added for different groups. Images were taken at 0 h, 24 and 48 h of incubation after the wound, respectively, for the average of wound closure measurement [17]. The wound boundaries were defined by applying ImageJ (version 1.52a; National Institutes of Health, USA) as described in our previous study [15].

### Transwell invasion assay

The MDA-MB-231 and BT-549 cells (1 × 10^4^) were seeded in each upper layer of culture insert of 3.0 µM pore size transell chamber (Falcon, USA) with the 100 µL medium containing 20% Matrigel (Corning, USA), 40 µM *TS* (or DMSO), and 0.1% FBS for each well. Under the cell permeable membrane, 600 µL of the complete medium was added for each chamber. After 24 h incubation, cells which have migrated through the membrane were fixed by 4% paraformaldehyde and dyed with crystal violet. The surface of upper layer of membrane was cleaned softly by neat cotton swabs, and the cells in different fields of view on the subface were counted by using ImageJ (version 1.52a; National Institutes of Health, USA) with triplicate.

### 3D spheroid formation assay

For evaluating the effect of *TS* on proliferation of TNBC cells, 3D spheroid formation assay was performed as described elsewhere [17]. The working concentration of *TS* was 40 µM. One set was used for capturing images from 24 to 96 h incubation time, and another set was for in vitro bioluminescence signal determination by transferring to a 96-well plate in the presence of D-luciferin (150 µL/mL) (PerkinElmer, USA). The proliferation inhibition rate was calculated following the formula as: inhibition rate = (1-Absorbance of treated sample /Absorbance of control sample )×100%.

### Mouse models and in vivo fluorescence imaging

Six-week female athymic nude mice (18–21 g) (n = 8) were purchased from Gempharmatech (Nanjing, China). Experimental protocols were approved by the Beijing University of TCM Institutional Animal care and Use Committee (No.104,195,489,042) and adhered to the NIH Guide for the Care and Use of Laboratory Animals. A freshly prepared mixture (50 µL complete culture medium, 5 × 10^5^ MDA-MD-231^Luc^, 50 µL Corning Matrigel Matrix HC, Phenol Red-free, and LDEV-free) was directly injected into lower right mammary fat pad (MFP) of each mouse under inhalation isoflurane anesthesia [15, 18, 19]. All mice were then maintained for 7 days and examined by In Vivo Imaging System (IVIS) Lumina LT Imaging System (Xenogen/Caliper Life Sciences, USA) with Living Image 4.3.1 software (Caliper Life Sciences, USA). All mice were then divided into *TS* group (n = 4) and NC group (the same final concentration of DMSO) (n = 4). *TS* was started to orally administer at 45 mg/kg body weight to mice once every 2 days. Images of tumor sizes were collected every week. Mice were monitored for 50 days to collect survivorship data. Mice were sacrificed in a CO_2_ chamber when ethically necessary due to clinical symptoms or substantial loss in body weight. To compare survival differences between *TS* treated and control groups, log rank (Mantel-Cox) test was used for survival analysis in GraphPad Prism (version 9.1.1).

### Statistical analysis

Each assay was performed at least 3 independent experiments with triplicate. Means and standard deviations were calculated, and the differences between groups were analyzed using a generalized linear model with post-hoc Tukey test for the correction of multiple comparisons. The two-tailed unpaired student’s t-tests were used for the difference comparison of two groups. Statistical significance was considered when P < 0.05 (two-sided). Kaplan-Meier Survival curve with a log-rank test was applied for survival curves.

## Results

### CAXII is overexpressed in TNBC and has a negative correlation with overall survival of TNBC patients

**Fig. 1:**
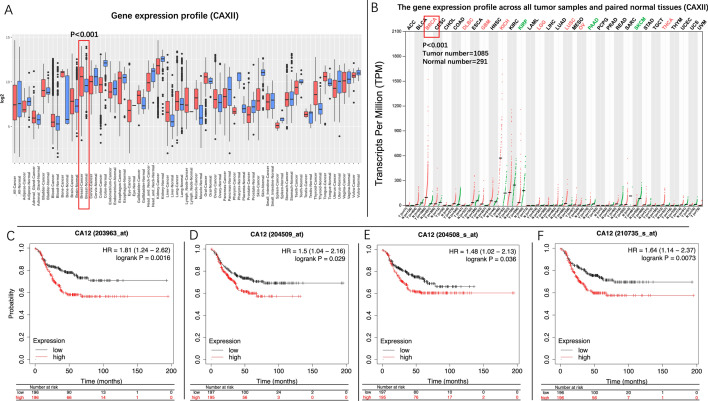
Gene profile overview analysis of ***CAXII***, and Kaplan–Meier survival curves of ***CAXII*** in TNBC. The expression of *CAXII* in breast cancer samples is significantly higher than of breast normal tissue (**A**, **B**) (P < 0.001). TNBC patients with a high expression level of *CAXII* had an inferior overall survival compared to those with a low level (P_203963_at_=0.0016, P_204508_s_at_=0.029, P_204509_at_=0.036, P_210735_s_at_=0.0073, Sample size: 392 in total) (**C**–**F**)

**Fig. 2:**
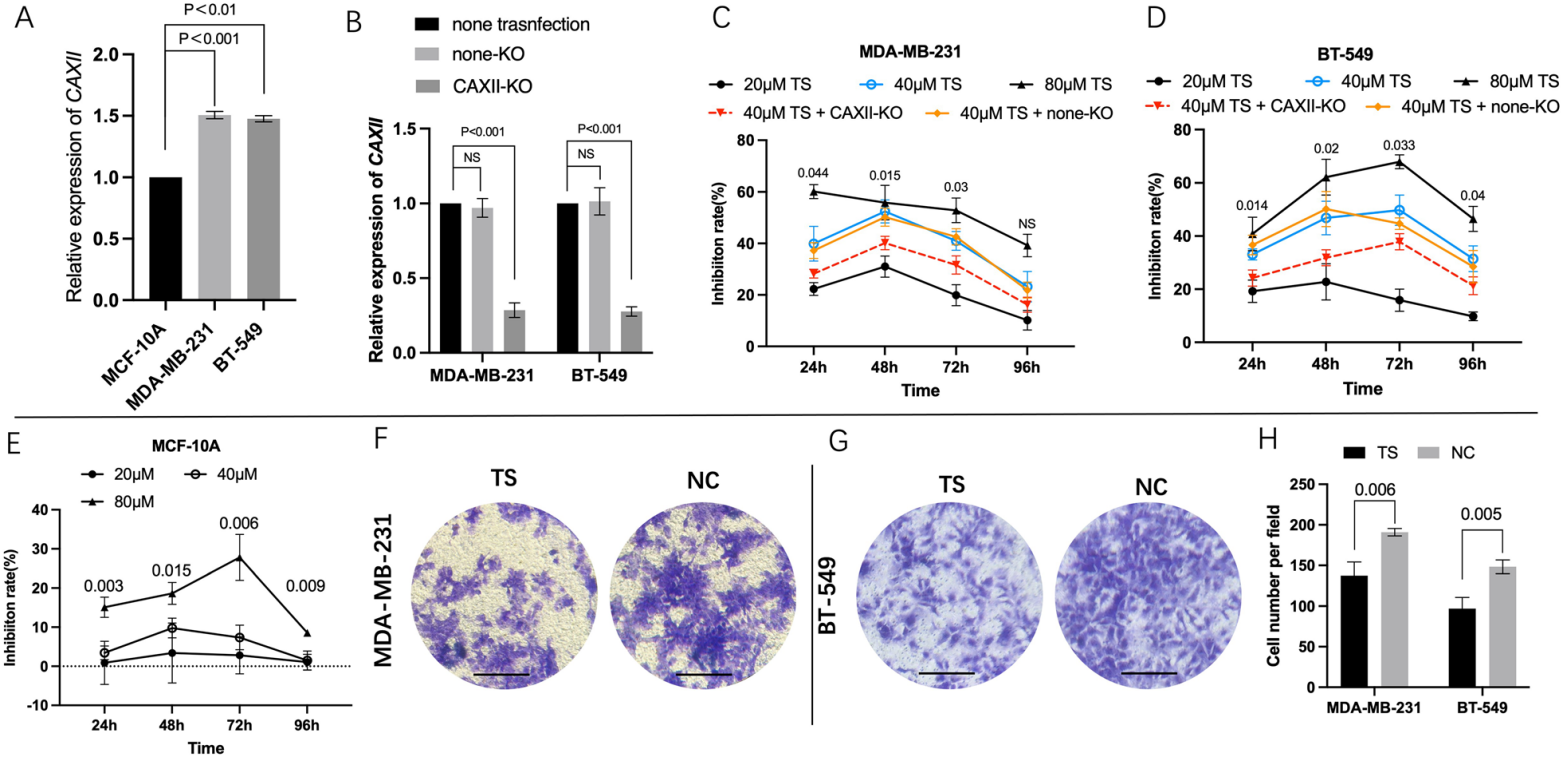
***TS*** inhibited the proliferation and invasion of MDA-MB-231 and BT-549 cells and the suppression by *CAXII* knockout. The relative *CAXII* expression in MCF-10 A was significantly lower than that of MDA-MB-231 and BT-549, respectively (P < 0.001) (**A**). Meanwhile its expression was significantly reduced in *CAXII*-KO group than non-KO and none transfection groups, in tested cells, respectively (P < 0.001) (**B**). Relative inhibition rates of MDA-MB-231, BT-549 and MCF-10 A treated by different concentrations of *TS*<SimplePara></SimplePara> were assessed by comparing the NC, respectively (**C**−**E**) (P values displayed in Fig. 2 C and 2D were calculated by comparing the inhibition rates of 40 µM *TS* group and 40 µM *TS* + CAXII-KO group). The average of cell number was counted by 3 randomly chosen different fields in both cell lines (Magnification 10×) (**F**–**H**). Values represent the mean ± SD. *NS* non-significant, *NC* negative control

The gene profile overview and survival analysis were performed at first. As shown in the gene expression profile analysis of GENT2, the expression of *CAXII* in breast cancer samples was significantly higher than breast normal tissue (P < 0.001). The analysis performed in GEPIA also displayed a similar result that the expression level of *CAXII* in breast invasive carcinoma (BRCA) samples was significantly higher than breast normal tissues (Fig. 1 A and B) (P < 0.001). Furthermore, the Kaplan–Meier survival curves showed that TNBC patients with a high level of *CAXII* (presented by red line) suffered worse prognosis than those with a low one (presented by black line) (HR = 1.81, 95% CI: 1.21–2.62, P < 0.001) (203963_at)(sample size: 392 in total). Similar trends were also observed in the other array probes (204508_s_at, 204509_at, 210735_s_at) (Fig. 1 C-1 F). Meanwhile, our RT-qPCR analysis also confirmed that the expression of *CAXII* in MDA-MB-231 was 1.51 ± 0.024 folds (P < 0.001), and in BT-549 was 1.48 ± 0.02 folds compared to MCF-10 A (P < 0.001) (Fig. 2 A).

### Expression of CAXII effects the anti-proliferation ability of *TS* on TNBC cells

As displayed in RT-qPCR results, in both MDA-MB-231 and BT-549 cells, the relative expression level of *CAXII* in CAXII-KO (*CAXII* knockout) group was significantly decreased than the parent cells and non-transfection groups (P < 0.001), respectively (Fig. 2B).

In MTS assay, MDA-MB-231, BT-549, and normal cell line of MCF-10 A were treated with either *TS* or DMSO, respectively (The groups represented as 20 µM *TS*, 40 µM *TS* and 80 µM *TS* were all conducted in non-transfection cells). An obvious dose-effect of relationship was observed. For MDA-MB-231 cells, at 48 h, the inhibition rates of 40 µM *TS* group was 52.44 ± 3.63%, which was significantly higher than that of 40 µM *TS* + CAXII-KO group (CAXII-KO cell treated by 40 µM *TS*) (P = 0.015), and was also significantly higher than that of 20 µM *TS* group (31.01 ± 3.33%, P = 0.004). Similar results were detected at 24 and 72 h. For BT-549 cells, the inhibition rates in 40 µM *TS* group were significantly higher than that of 40 µM *TS* + CAXII-KO group and 20 µM *TS* group, respectively, during the whole observation period (P < 0.05). For both cells, inhibition rates in 40 µM *TS* group showed non-significant difference compared to their counterparts in 40 µM *TS* + non-KO group (Fig. 2 C and D). However, the inhibition rates just ranged from 1.48 ± 1.96% to 9.78 ± 2.05%, during the whole period (Fig. 2E).

### *TS* suppresses invasion, migration and 3D formation of TNBC in vitro

The invasion assay showed that, in MDA-MB-231 cells, the invasive cell number per field in *TS* group (137.67 ± 13.47) was significantly lower than that of NC group (191 ± 3.74, P = 0.006). A similar result was observed in BT-549 cells (Fig. 2 F, G and  H).

**Fig. 3:**
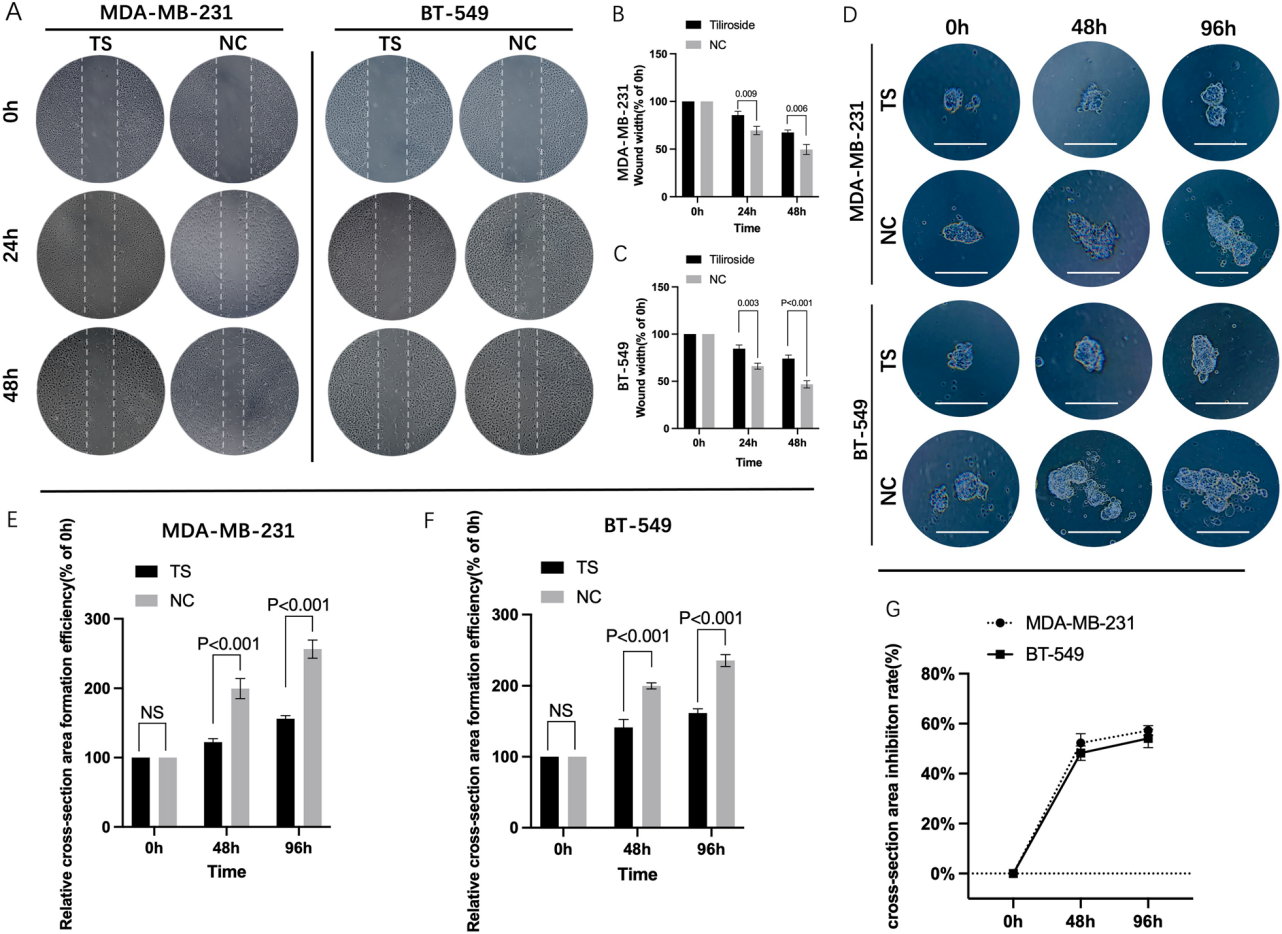
***TS*** inhibited the migration and 3D formation abilities of TNBC cells. The wound healing assay was performed in MDA-MB-231 and BT-549 cells (**A**). The bar graphics present the percentage of wound recovery in both cell lines, respectively. In MDA-MB-231, the average width of wound gaps in the *TS* group was 85.66 ± 3.26% of its initial width compared to its counterpart in NC group (69.57 ± 3.56%, P = 0.009), at 24 h, and its 67.39 ± 2.14% compared to 49.52 + 4.29%, at 48 h (P = 0.006) (**B**). Similar results were detected in BT-549 cells (**C**). The representative 3D spheroid models of TNBC cells (**D**). In MDA-MB-231 cells, the relative cell cross-section area of *TS* group was 122.33 ± 4.03% compared to199.67 ± 11.9% of NC group, at 48 h (P < 0.001), and 156 ± 3.74% compared to 256.27 ± 10.73% at 96 h (P = 0.006) (**E**). Similar trends were also observed in BT-549 cells (**F**). The inhibition rates of MDA-MB-231 cells ranged from 52.29 ± 3% to 57.24 ± 1.61%, at 48 and 96 h, and that of BT-549 were 48.21 ± 2.36% to 54.1 ± 3%, by calculating the fluorescence values of 3D formation cell (**G**). NC negative control

The cell migration assay displayed that the tested TNBC cells treated by *TS* healed slower than those in NC groups (Fig. 3 A). For MDA-MB-231, the average width of wound gaps in the *TS* group was 85.66 ± 3.26% of its initial width compared to its counterpart in NC group (69.57 ± 3.56%, P = 0.009), at 24 h. The width in the *TS* group shrunk to 67.39 + 2.14%, while its counterpart in the control group healed to 49.52 + 4.29% (P = 0.006), at 48 h. Similar results were detected in BT-549 cells (Fig. 3B C).

3D spheroid formation assay was also conducted in MDA-MB-231 and BT-549 cells which contained a luciferase reporter gene. The dynamic changes of 3D spheroid formation are shown in Fig. 3D. For MDA-MB-231 cells, at 48 h, the relative cell cross-section area of *TS* group grew to 122.33 ± 4.03% of its initial area, and significantly lower than 199.67 ± 11.9% of NC group (P < 0.001). At 96 h, the formation efficiency of *TS* group reached to 156 ± 3.74% compared to 256.27 ± 10.73% of NC group (P = 0.006). Similar trends were also observed in BT-549 cells (Fig. 3E F). In addition, the luciferase reporter gene assays displayed that the inhibition rates of MDA-MB-231 cells ranged from 52.29 ± 3% to 57.24 ± 1.61%, at 48 and 96 h, and that of BT-549 were 48.21 ± 2.36% and 54.1 ± 3%(Fig. 3G).

### ***TS*** suppresses activity and expression of CAXII and regulates *E2F1/3/Caspase-3* axis

**Fig. 4:**
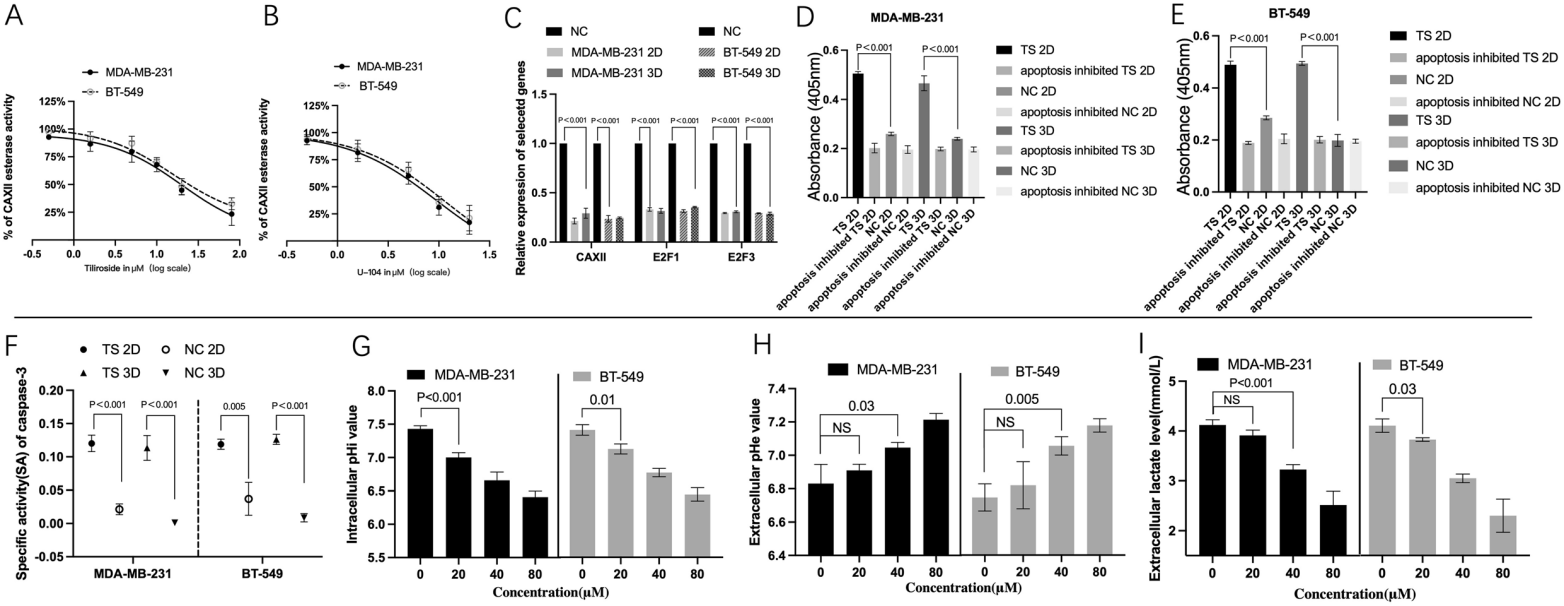
*TS* regulated the activity, expression of *CAXII*, ***E2F1,3***/Caspase-3 axis and pHi, pHe and extracellular lactate. Dose-dependent inhibition curves of CAXII esterase activity under different intervention were displayed s in  **A** and** B**. For *TS* group, the IC50 was 20.95 ± 5.8 µM in MDA-MB-231 cells and 30.92 ± 6.98 µM in BT-549 cells (**A**), and that in U-104 group were 5.47 ± 1.25 µM and 6.62 ± 1.8 µM, respectively (**B**). As RT-qPCR results showed, in 2D cultured MDA-MB-231 cells, the expression level of *CAXII* was 0.21 ± 0.024 folds compared to its NC (P < 0.001) after the intervention of *TS*, and that was 0.316 ± 0.02 folds of NC (P < 0.001) in 3D *TS* group. Similar results were observed in rest groups (**C**). As for Caspase-3 activity assay, in MDA-MB-231 cells, the value of *TS* group 2D (0.50 ± 0.01) was significantly higher than that of apoptosis inhibited *TS* group 2D (0.20 ± 0.02, P < 0.001). In 3D system, the value of *TS* group 3D (0.47 ± 0.02) was also significantly higher than that of apoptosis inhibited *TS* group 3D (0.19 ± 0.01, P < 0.001). Similar results were observed in BT-549 cells (**D** and** E**). Furthermore, calculated CASP3-specific activity in *TS* group 2D (0.12 ± 0.01) was significantly higher than that in NC group 2D (0.03 ± 0.01, P < 0.001). Similar result was also detected 3D cultured MDA-MB-231 and BT-549 cells (**F**). For MDA-MB-231 cells, the intracellular pHi value of 0 µM *TS* group (7.43 ± 0.04) was significantly higher than that of 20, 40, 80 µM *TS* group, respectively (7.00 ± 0.06, 6.66 ± 0.1, 6.41 ± 0.08, P < 0.001). Similar results were observed in BT-549 cells in extracellular lactate level (G and I). Opposite trends were displayed in extracellular pHe for both cells(H),* NC* negative control

CAXII esterase activity was measured after 1 h intervention of the same amount of *TS* or U-104 (as a positive control). As shown in Fig. 4 A and B, the activity curves were in a dose-dependent manner. For *TS* group, the IC50 was 20.95 ± 5.8 µM in MDA-MB-231 cells and 30.92 ± 6.98 µM in BT-549 cells. In addition, the activities were 5.47 ± 1.25 µM and 6.62 ± 1.8 µM for MDA-MB-231 and BT-549, under the intervention of U-104 (Fig. 4 A and 4B).

RT-qPCR results displayed that in MDA-MB-231 cells, the expression level of *CAXII* in 2D cultured cells after *TS* intervention was 0.21 folds compared to its NC (P < 0.001), and in 3D *TS* group was 0.316 folds of NC (P < 0.001). For gene *E2F1*, its expression in 2D *TS* group was 0.33 folds of NC (P < 0.001), and in 3D *TS* group was 0.316 folds of NC (P < 0.001), respectively. Similar results were found in BT-549 cells for *E2F3* (Fig. 4 C).

As shown in caspase-3 activity assay, in MDA-MB-231 cells, the absorbance of *TS* group 2D was significantly higher than that of apoptosis inhibited *TS* group 2D (0.50 vs. 0.20, P < 0.001). In 3D system, the absorbance of *TS* group 3D was also significantly higher than that of apoptosis inhibited *TS* group 3D (0.47 vs. 0.19, P < 0.001). Similar results were observed in BT-549 cells cultured in either 2D or 3D systems (Fig. 4D and E). Moreover, as for MDA-MB-231 cells, calculated CASP3-specific activity in *TS* group 2D was significantly higher than that in NC group 2D (0.12 vs. 0.03, P < 0.001). Their counterparts in 3D system also showed significant difference between *TS* group and NC group, (0.11 vs. 0.001, P < 0.001). Similar result was also detected in BT-549 cells (Fig. 4 F).

### Effects of* TS* on the levels of pHi, pHe and extracellular lactate

Detections of intracellular pH (pHi), extracellular pH (pHe) and lactate level were also performed. As shown in Fig. 4G, in MDA-MB-231 cells, the intracellular pHi value of 0 µM *TS* group was 7.43 ± 0.04 compared to 7.00 ± 0.06 of 40 µM *TS* group (P < 0.001). Intracellular pHi value of 40 µM *TS* group dropped to 6.66 ± 0.1, and further to 6.41 ± 0.08 in 80 µM *TS* group. Similar results were observed in BT-549 cells. In contrast, in MDA-MB-231 cells, the extracellular pHe value in 40 µM *TS* group (7.05 ± 0.02) or 80 µM *TS* group (7.21 ± 0.03) was significantly higher than that of 0 µM *TS* group (6.83 ± 0.1), respectively. Similar results were observed in BT-549 cells (Fig. 4 H). For an extracellular lactate level, a significant dose-dependent relationship was observed in BT-549 cells (from 4.1 ± 0.11 of 0 µM *TS* group to 2.3 ± 0.27 of 80 µM *TS* group). However, in MDA-MB-231 cells, compared with NC group (4.12 ± 0.09), the significant reduction of extracellular lactate level started to reveal when the concentration of *TS* reached 40 µM (3.23 ± 0.08, P < 0.01) (Fig. 4I).

### Anticancer Effect of ***TS*** in Xenograft Mouse Models

**Fig. 5:**
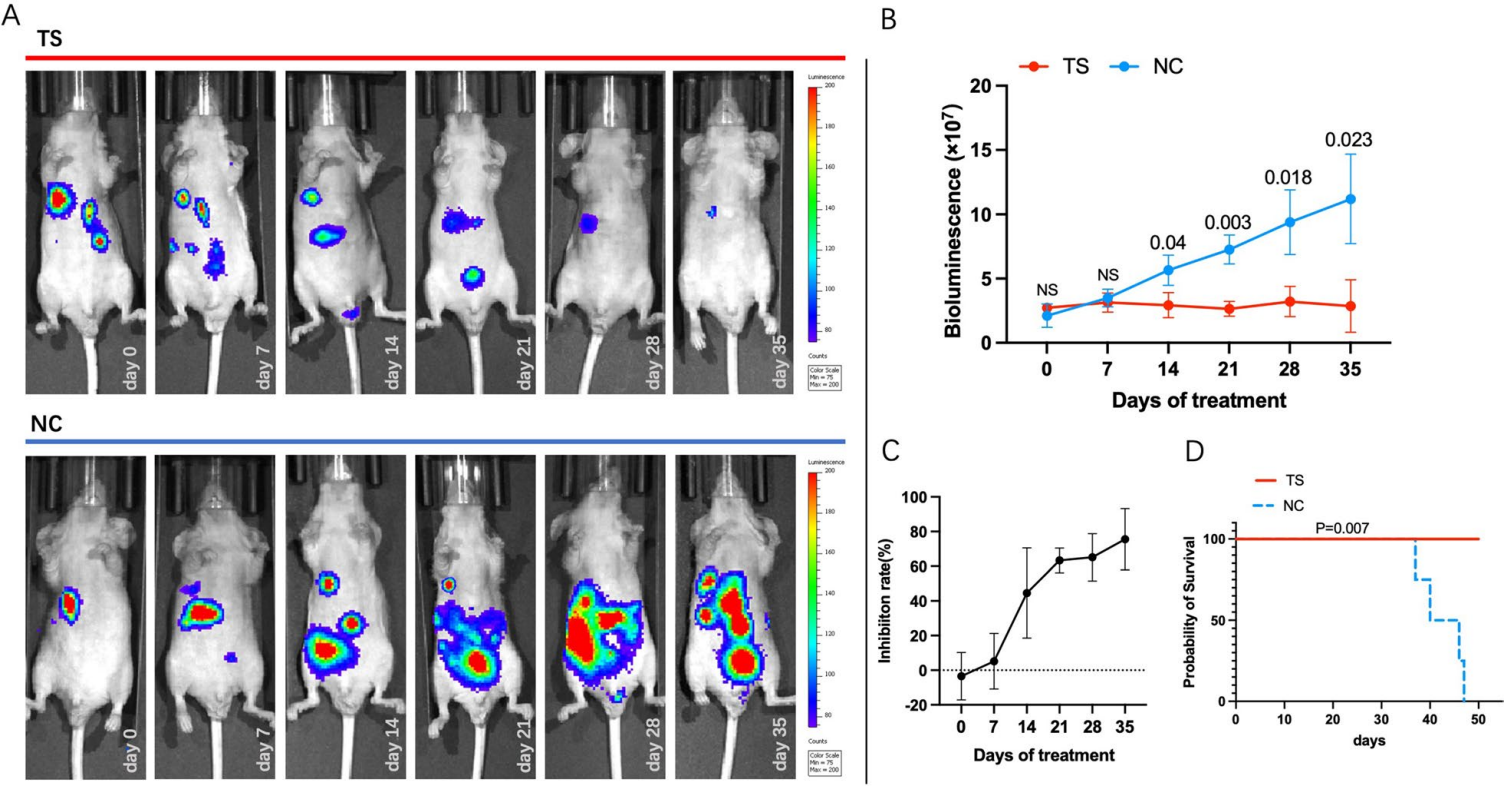
*TS* suppressed the development of TNBC in vivo. The images of bioluminescence of TNBC implanted in xenograft mice of MDA-MB-231^luc^, from 0 to the 35th day of treatment, were displayed (*TS* was given at 45 mg/kg body weight to mice once every 2 days orally) (**A**). At 14th day, the bioluminescence value in *TS* group ((2.93 ± 0.8)×10^7^) was significantly lower than that of NC ((5.65 ± 0.96) ×10^7^, P < 0.05), and this trend remained till the 35th day of treatment (**B**). The inhibition rate was 5.17 ± 13.11% at 7th day, and gradually climbed to 75.53 ± 14.45% at 35th day of treatment (**C**). As for survival assay, mice in *TS* group were all alive by the end of the 50th day of treatment, and mice of NC group all died before 48th day of treatment (log-rank P = 0.007) (D)

Tumor-bearing mouse model was also used to further evaluate the effect of *TS* on TNBC cells. The represented bioluminescence tumor images of tested mice were shown in Fig. 5 A. The tumors of *TS* treated mice started to show significant difference compared to NC group at 14th day of treatment. Briefly, the bioluminescence of *TS* group (2.93 ± 0.8)×10^7^ was significantly lower than that of NC group (5.65 ± 0.96) ×10^7^, at 14th day of treatment (P = 0.04). At 35th day of treatment, this trend still remained, which was (2.86 ± 1.67) ×10^7^ of TC compared to (11.2 ± 2.84) ×10^7^ of NC (P = 0.023) (Fig. 5B). The inhibition rate based on tumor bioluminescence signal was 5.17 ± 13.11% at 7th day of treatment, and 75.53 ± 14.45% at 35th day of treatment (Fig. 5 C). Mice in *TS* group were all alive by the end of the 50th day of treatment. However, 4 mice of NC group died at 37th, 40th, 46th and 47th day of treatment, respectively (log-rank P = 0.007) (Fig. 5D).

## Discussion

The addition of new “bullets” in the treatment of breast cancer may accelerate the cure of the disease, given that the combination of different regimens or therapies substantially prolongs breast cancer survivors. *TS* (TS), a compound from several plants such as Tribulus terrestris and Agrimonia pilosa ledeb [20], belongs to a saponin of the herb. Herbal saponin was reported to possess therapeutic effects against cardiovascular diseases, nervous system disorders, asthma, arthritis and diabetes [21]. Our previous study has reported the anti-cancer effects of *TS* on hepatocellular carcinoma as a CAXII inhibitor [9]. In this preclinical study, we extended our previous study and showed its inhibitive effects against TNBC.

Carbonic anhydrases (CA) catalyze the reversible hydration of carbon dioxide to bicarbonate and protons. Overexpression of CAXII (CA12) has been found in hypoxia—the oxygen deprivation of cancer cells due to a combination of poor tumor vascularization and high proliferation rate, despite the fact that hypoxia response elements (HREs) essentially lack in the 5’-upstream genomic region of CAXII gene. Moreover, overexpression of transmembrane CAXII in cancer has been found to be associated with cancer progression including rapid tumor growth and invasion, infiltration of surrounding normal tissues and the formation of metastases [22]. Moreover, selectively silencing of CAXII gene in TNBC cell line MDA-MB-231 could lead to the supression of cell migration and invasion by interefering the p38 mitogen-activated protein kinases (p38 MAPK) signalling pathway [10]. Similarly, the reduction of CAXII expression by blocking Hedgehog signaling pathway in MDA-MB-231 cells could limit the migration ability of cancer cells, which highlighted the potential role of CAXII to be an effective therapeutic target in TNBC treatment.

In line with the previous studies, in this study, we found that expression of *CAXII* in clinical breast-cancer samples was significantly higher than in breast-normal tissues (Fig. 1A and B). Such phenomenon is also reproducible in our RT-qPCR assay that TNBC cells (MDA-MB-231 and BT-549) possess significantly higher level of *CAXII* expression than in breast normal cell (MCF-10 A) (Fig. 2A). In addition, a worse overall survival was also found in patients with high expression of *CAXII*. Furthermore, we also applied U-104 (SLC-0111), a potent specific carbonic anhydrase (CA) inhibitor for CAXII, to evaluate the inhibitive specificity of *TS* as a positive control, as we did in our previous study[9]. In both MDA-MB-231 and BT-549 cells, CAXII esterase activity assay showed that *TS* could suppress CAXII esterase activity in a dose-dependent manner similar to the U-104 groups, which further revealed the inhibitive effect of *TS* on CAXII. These observations suggest that CAXII is a potential therapeutic target, and may be exploited for the treatment of TNBC.

Cellular acidosis was reported to be one of triggers of apoptosis, therefore a high level of pHi was thought to prevent cancer cells from apoptosis. Evidence has also displayed that pHi reduction can cause cellular apoptosis by activating endonuclease II and MMP impairment [23]. Furtheremore, caspase activation and mitochondrial depolarisation have been found to be activated by cytochromic acidification, staurosporine and ultraviolet light. Therefore the agents, including CAXII inhibitor, which can reduce the pHi in cancer cells, were thought to be pro-apoptotic by impairing intracellular homeostasis and metabolism of cancer cell [24]. Additionally, the accumulation of the metabolic byproduct lactate and extracellular acidification have been reported to accelerate tumor cell proliferation, metastasis, and angiogenesis. Lactate acidosis has been found to reduce immune evasion in TNBCs [25, 26]. Therefore, extracellular lactate reduction has been considered as an anti-cancer approach to enhancing the therapeutic effect of immune therapy[27]. CAXII has been reported to maintain pH and CO_2_ homeostasis, thereby affecting cancer progression, invasion, and resistance to therapy [8]. In this study, it is the first time to display that *TS* could not only significanly reduce the gene expression and activity of CAXII, but also increased pHe level and suppressed extracellular lactate and pHi levels, which creates a hostile microenviroment to block TNBC progressions. It also indicates that *TS* possesses regulatory capacity for cellular acidosis which specifically possessed by CAXII inhibitors [8].

*TS* showed significantly strong inhibitive effects on the proliferation, invasion and 3D spheroid formation of two TNBC cell lines, while had a limited impact on the normal cells. As for in vivo experiments, our results show that mice receiving *TS* treatment experienced a significantly decreased tumor burden (> 40% after 14d), and also had a significantly improved survival rate, indicating the efficacy of *TS* in TNBC treatment. The results of 3D formation assay, and intracellular or extracellular PH values testing assays indicate that *TS* could shape an effective antitumor microenvironment.

*E2F1* and *E2F3* are oncogenes and have negative correlations with breast cancer patient survival [17]. *E2F1* has been reported to affect cell growth by regulating NF-κB, thereby enhancing tumor proliferation and anti-apoptosis. It can also cause immune escape by blocking the transcription of ICAM-1 [28]. Meanwhile, *E2F3* expression appears to provide a growth advantage to tumor cells by activating cell proliferation [29]. Overexpression of *E2Fs* is frequently observed in advanced cancers and aggravates chemoresistance. Overexpress of *E2F1* and *E2F3* were found in breast adenocarcinoma when compared to normal breast tissues [17]. In addition, Caspase3 (CASP3), as an apoptosis executive factor, not only can regulate many physiological processes including cancer stemness and autophagy, but also affect pyroptosis by mediating GSDM [30]. Lack of CASP3 can lead to the resistance of cells to microenvironmental stress and treatments, thereby promoting tumorigenesis [31]. Our previous studies have shown that *E2F1/E2F3*/Caspase-3 axis, which is associated with multiple cellular physiological processes, such as cancer stemness and apoptosis, was an effective anti-cancer pathway in liver cancer and breast cancer (including TNBC), and that *TS* had a positive effect on regulating such axis in HCC [9, 15, 17]. Again, in this study, we demonstrated that *TS* significantly inhibited the expression of *E2F1* and*E2F3*, and increased CASP3 activity in both TNBC cell lines cultured in 2D and 3D systems.

Interestingly, we also found that *TS* as CAXII inhibitor also could reduce the expression of CAXII, E2F1/E2F3 expression. This finding suggests that a positive feedback loop may exist. It has been shown that low pH levels under hypoxia conditions could induce CA expression [7], and that acidic microenvironments could stimulate E2F pathway to promote tumor metastasis [32, 33].

Limitations exist in this study. We uncovered the anti-cancer effects of *TS* on TNBC in vitro and in vivo with a focus on targeting CAXII only. Since isoforms exist for CA, and one of which the isoform CAII ranks the third predicted targets of *TS*, we cannot rule out the possibility of inhibitive effect of *TS* on CAII, as a cancer malignant behavior regulator [9, 34, 35]. Therefore, it will be interesting to further investigate the expression profile of other CA isoforms and relevant molecular docking in future studies for further digging the potential mechanisms of anticancer effects exerted by *TS*. Moreover, in addition to further screening for more optimal working concentration, nanoparticles should also be applied to further avoid the risk of potential cytotoxicity of *TS* in future study. Our previous study has shown that nanoparticle LNP-DP1 efficiently alleviated the cytotoxicity of *TS* for normal cells [15]. To further confirming and evaluating the anti-metastasis potential of *TS* on TNBC cells, multiple positions of IVIS scanning combined with histological or genetic analysis of metastatic lesions shall be investigated in future study [36].

In summary, we demonstrate that *TS* suppresses TNBC development by acting as a novel CAXII inhibitor in this study. To our knowledge, this is the first study to show the inhibitive effect of *TS* on TNBC, low-toxic property of TS on normal breast cell and the regulation of *TS* on *E2F1*/*E2F3*/Caspase-3 axis. Our results indicate a novel chemotherapeutic *TS* with CAXII functionality as a potential choice for TNBC treatment. Further investigation of *TS* is warranted to explore its therapeutic potential for the treatment of TNBC and other potential anti-cancer pathways.

## Data Availability

The data supporting the results of this study are publicly available from Figureshare at 10.6084/m9.figshare.21398841.v1.
